# A Bearing Fault Diagnosis Method Based on PAVME and MEDE

**DOI:** 10.3390/e23111402

**Published:** 2021-10-25

**Authors:** Xiaoan Yan, Yadong Xu, Daoming She, Wan Zhang

**Affiliations:** 1 School of Mechatronics Engineering, Nanjing Forestry University, Nanjing 210037, China; 2 School of Mechanical Engineering, Southeast University, Nanjing 211189, China; ydxu@seu.edu.cn; 3 School of Mechanical Engineering, Jiangsu University, Zhenjiang 212013, China; 1000005461@ujs.edu.cn; 4 Department of Automation, Nanjing University of Information Science and Technology, Nanjing 210044, China; zhangwan@nuist.edu.cn

**Keywords:** variational mode extraction, multiscale envelope dispersion entropy, rolling bearing, fault diagnosis

## Abstract

When rolling bearings have a local fault, the real bearing vibration signal related to the local fault is characterized by the properties of nonlinear and nonstationary. To extract the useful fault features from the collected nonlinear and nonstationary bearing vibration signals and improve diagnostic accuracy, this paper proposes a new bearing fault diagnosis method based on parameter adaptive variational mode extraction (PAVME) and multiscale envelope dispersion entropy (MEDE). Firstly, a new method hailed as parameter adaptive variational mode extraction (PAVME) is presented to process the collected original bearing vibration signal and obtain the frequency components related to bearing faults, where its two important parameters (i.e., the penalty factor and mode center-frequency) are automatically determined by whale optimization algorithm. Subsequently, based on the processed bearing vibration signal, an effective complexity evaluation approach named multiscale envelope dispersion entropy (MEDE) is calculated for conducting bearing fault feature extraction. Finally, the extracted fault features are fed into the k-nearest neighbor (KNN) to automatically identify different health conditions of rolling bearing. Case studies and contrastive analysis are performed to validate the effectiveness and superiority of the proposed method. Experimental results show that the proposed method can not only effectively extract bearing fault features, but also obtain a high identification accuracy for bearing fault patterns under single or variable speed.

## 1. Introduction

Rolling bearings are one of the important parts of mechanical transmission system, which plays an extremely important role in wind power generation, rail transportation, petrochemical engineering and other modern industries [1]. Due to the influence of the harsh and high strength working environment, bearings are prone to various failures (e.g., inner race, outer race and ball fault). If a bearing local fault cannot be detected in a timely manner, it will pose a serious threat to personal safety and have a significant impact on social and economic development [2]. Therefore, the high-efficiency fault diagnosis of rolling bearings has the important practical significance for keeping a mechanical equipment in good condition.

Because the practical bearing vibration signal has strong nonstationary and nonlinear traits, traditional methods are very difficult to address this kind of problem. Hence, many signal processing techniques have been presented to analyze and process the nonstationary and nonlinear bearing vibration signal, such as empirical mode decomposition (EMD) [3], empirical wavelets transform (EWT) [4], local mean decomposition [5], adaptive local iterative filtering (ALIF) [6], symplectic geometry mode decomposition (SGMD) [7], variational mode decomposition (VMD) [8], successive multivariate variational mode decomposition (SMVMD) [9], the improved variational mode decomposition based on fractional Fourier transform (VMD-FRFT) [10] and so on. The above mentioned methods have been successfully used in mechanical vibration signal processing and bearing fault diagnosis. Kostopoulos [11] adopted EMD and Hilbert-Huang transform to extract bearing fault features and applied the hybrid ensemble detector to identify bearing health conditions. Yu et al. [12] employed EMD and principal component analysis (PCA) to extract and select damage-sensitive features. Zhao et al. [13] proposed an enhanced empirical wavelet transform (MSCEWT) based on a maximum-minimum length curve method to diagnose the fault types of motor bearings. Liu et al. [14] proposed a time-frequency representation method based on robust local mean decomposition to analyze multicomponent amplitude-modulated and frequency-modulated signal and execute bearing fault diagnosis. Zhang et al. [15] combined the k-optimized adaptive local iterative filtering, improved multiscale permutation entropy and BP neural network to achieve fault classification of rolling bearings. Zheng and Xin [16] used symplectic geometry mode decomposition (SGMD) and power spectral entropy (PSE) to extract fault feature information of a hydraulic pump signal. Jiang et al. [17] employed VMD and a multiresolution teager energy operator to extract the fault-related impulses hidden in the raw bearing vibration signal. Among the above methods, due to the solid theoretical foundation, strong noise robustness and good antimodal aliasing ability, the application of VMD is most frequent in bearing fault diagnosis. Nevertheless, VMD suffers from two serious problems [18]. Firstly, the computational efficiency of VMD is relatively slow, which is not conducive to online monitoring. Secondly, the performance of VMD is largely determined by its two input parameters (i.e., the penalty factor and the number of decomposition mode). Focus on these problems, a new signal processing method named variational mode extraction (VME) is proposed by Nazari and Sakhaei [19] in 2018, which can avoid the disadvantages of high computational burden existing in the VMD method. However, similar to VMD, in the VME method, there are also two key parameters (i.e., the penalty factor and mode center-frequency) that need to be artificially selected [20]. Therefore, to solve this issue, this paper proposes a parameter adaptive variational mode extraction (PAVME) to process the collected bearing vibration data by introducing a new parameter optimizer called whale optimization algorithm (WOA) to automatically and effectively determine the important parameters (i.e., the penalty factor and mode center-frequency) of VME.

According to the fault diagnosis process of rolling bearings, after vibration signal processing using the VME method, the effective bearing fault feature extraction is critical for obtaining a good fault diagnosis result. Currently, entropy-based feature extraction has attracted more and more attention in bearing fault diagnosis. Common entropy methods have spectral entropy [21], sample entropy (SE) [22], permutation entropy (PE) [23], fuzzy entropy (FE) [24], Deng entropy [25], symbolic entropy [26] and dispersion entropy (DE) [27]. However, these entropies only extract bearing fault information at a single scale. Hence, to extract more fault information over multiple scales, their multiscale versions (e.g., multiscale sample entropy (MSE) [28], multiscale permutation entropy (MPE) [29], multiscale fuzzy entropy (MFE) [30] and multiscale dispersion entropy (MDE) [31]) are also developed for evaluating the complexity of a time series and revealing fault characteristic information hidden in bearing vibration signal. Among these multiscale entropies, the performance of MSE and MPE are influenced by data length, that is, they are easy to generate the undefined entropy value for short-term time series. Compared with MSE and MPE, MDE has less dependence on data length and faster running speed [32]. When rolling bearing has a local fault, there are a series of periodic impulse trains in the resulting bearing vibration signal, the envelope demodulation method has been shown to be effective in excavating periodic impulse feature information [33]. Therefore, considering the advantages of MDE and envelope demodulation, this paper proposes a new signal complexity evaluation method named multiscale envelope dispersion entropy (MEDE) by integrating the envelope signal into MDE, which can more accurately describe complexity and uncertainty of a time series. In a word, the main contributions and novelties of this paper are summarized as follows:

(1) A new signal processing method named parameter adaptive variational mode extraction (PAVME) based on the whale optimization algorithm (WOA) is proposed, which can avoid the shortcomings of empirical parameter selection of the original VME. Concretely, the PAVME method is regarded as a preprocessor to process the original collected bearing vibration signal, which is aimed at removing some signal interference components and highlighting the frequency components related to bearing faults.
(2) A novel complexity index named multiscale envelope dispersion entropy (MEDE) is presented by combining envelope analysis and MDE. Specifically, MEDE is regarded as a feature extractor to extract the useful bearing fault feature information.
(3) A bearing fault diagnosis method based on PAVME and MEDE is proposed for automatically identifying bearing fault categories.
(4) The comparison and analysis of experimental cases validate the effectiveness and superiority of the proposed method in bearing fault identification.

The organization of this paper is as follows. Section 2 introduces the parameter adaptive variational mode extraction and conducts the comparison among PAVME, VME, VMD and EMD. Section 3 describes the theory of multiscale envelope dispersion entropy and conducts the comparison among MEDE, MDE, MPE and MSE. Section 4 shows the specific steps of the proposed fault diagnosis method. Section 5 validates the effectiveness of the proposed method by using experimental data analysis. Section 6 draws the conclusion part of this paper.

## 2. Parameter Adaptive Variational Mode Extraction

### 2.1. Variational Mode Extraction

Variational mode extraction (VME) is a new signal processing method, which can effectively obtain the desired mode components by presetting the penalty factor and mode center-frequency. The theoretical ideas of VME are similar to VMD, but it is faster than the VMD because it only looks for the specified frequencies. Briefly speaking, in the VME, the original time series f(t) can be split into two parts by the following equation:

(1) $$f(t)=u_{d}(t)+f_{r}(t)$$

where $u_{d}(t)$ is the desired mode components, $f_{r}(t)$ is the residual signal. Specifically, mode extraction process of VME is established based on the following three conditions.

(1) The desired mode components have compactness around the center-frequency. To achieve this goal, minimization problem of the following objective function is solved to obtain the desired compact mode components.

(2) $$J_{1}={\left\|\partial_{t}\left[\left(\delta (t)+\frac{j}{\pi t}\right)*u_{d}(t)\right]e^{-j\omega_{d}t}\right\|}_{2}^{2}$$

where $\omega_{d}$ denotes the center-frequency of mode components $u_{d}(t)$, δ(t) represents the Dirac distribution, and the asterisk * represents the convolution operation.

(2) Spectral overlap of the residual signal $f_{r}(t)$ and the desired mode components $u_{d}(t)$ should be as small as possible. That is, in the frequency band of the desired mode components, the energy of the residual signal $f_{r}(t)$ should be minimized. Particularly, when the energy of the residual signal $f_{r}(t)$ around the center-frequency is equal to 0, a complete and accurate mode component will be obtained. To overcome these limitations, the contents of the residual signal $f_{r}(t)$ are firstly found out via using a proper filter, and then the energy of the residual signal $f_{r}(t)$ is regarded as the indicator to evaluate the spectral overlap degree of $f_{r}(t)$ and $u_{d}(t)$. For this purpose, here a filter with frequency response of $\hat{\beta }(\omega )$ is designed:

(3) $$\hat{\beta }(\omega )=\frac{1}{\alpha {\left(\omega -\omega_{d}\right)}^{2}}$$

where $\hat{\beta }(\omega )$ is similar to the Wiener filter at the frequencies far away from $\omega_{d}$, this because it has an infinite gain at $\omega =\omega_{d}$. Hence, the following penalty function is adopted to minimize the spectral overlap of $f_{r}(t)$ and $u_{d}(t)$.

(4) $$J_{2}={\left\|\beta (t)*f_{r}(t)\right\|}_{2}^{2}$$

where β(t) denotes the impulse response of the designed filter.

(3) The obtained mode components $u_{d}(t)$ should be meet the equality constraint listed in Equation (1) to guarantee complete reconstruction. That is, the extraction problem of the desired mode components can be expressed as solving the following constrained minimization problem:

(5) $$\begin{array}{l} \;\;\;\;\;\;\;\;\underset{u_{d},\omega_{d},f_{r}}{\min}\{\alpha J_{1}+J_{2}\}\\ \begin{array}{cc} subject\;to: & u_{d}(t)+f_{r}(t)=f(t) \end{array} \end{array}$$

where α is the penalty factor of balancing $J_{1}$ and $J_{2}$. To solve the above reconstruction constrained problem, the following augmented Lagrangian function is adopted by introducing the quadratic penalty term and Lagrangian multiplier.

(6) $$\begin{array}{l} L(u_{d},\omega_{d},f_{r},\lambda ):=\alpha {\left\|\partial_{t}\left[\left(\delta (t)+\frac{j}{\pi t}\right)*u_{d}(t)\right]e^{-j\omega_{d}t}\right\|}_{2}^{2}\\ \;\;\;\;\;+{\left\|\beta (t)*f_{r}(t)\right\|}_{2}^{2}+{\left\|f(t)-\left(u_{d}(t)+f_{r}(t)\right)\right\|}_{2}^{2}\\ \;\;\;\;\;\;\;\;\;\;\;+\langle \lambda (t),f_{r}(t)-\left(u_{d}(t)+f_{r}(t)\right)\rangle \end{array}$$

where λ is the Lagrangian multiplier. According to the Parseval theorem, by using ω instead of $\omega -\omega_{d}$ and adopting the equality ${\left\|\hat{x}(\omega )\right\|}_{2}^{2}={\left\|\hat{x}(\omega -\omega_{d})\right\|}_{2}^{2}$, the above Equation (6) can be rewritten as follows:

(7) $$\begin{array}{l} L(u_{d},\omega_{d},f_{r},\lambda )=\alpha {\left\|j(\omega -\omega_{d})\left[\left(1+\mathrm{sgn}(\omega )\right){\hat{u}}_{d}(\omega )\right]\right\|}_{2}^{2}\\ \;\;+{\left\|\hat{\beta }(\omega ){\hat{f}}_{r}(\omega )\right\|}_{2}^{2}+{\left\|\hat{f}(\omega )-\left({\hat{u}}_{d}(\omega )+{\hat{f}}_{r}(\omega )\right)\right\|}_{2}^{2}\\ \;\;\;\;\;\;\;\;\;\;\;+\langle \hat{\lambda }(\omega ),{\hat{f}}_{r}(\omega )-\left({\hat{u}}_{d}(\omega )+{\hat{f}}_{r}(\omega )\right)\rangle \end{array}$$

To solve the minimization problem of augmented Lagrangian function, the alternate direction method of multipliers algorithm (ADMM) is introduced. In ADMM, multiple iteration suboptimizations are conducted to obtain the optimization variables ($u_{d}$, $\omega_{d}$, and $f_{r}$). Hence, in the *n* + 1 iteration, the mode components ${\hat{u}}_{d}^{n+1}$ can be obtained by the following equation:

(8) $$\begin{array}{l} {\hat{u}}_{d}^{n+1}\leftarrow \underset{u_{d}\in X}{\arg\min}\{\alpha {\left\|j(\omega -\omega_{d})\left[\left(1+\mathrm{sgn}(\omega )){\hat{u}}_{d}(\omega \right)\right]\right\|}_{2}^{2}+\\ \;\;+{\left\|\hat{\beta }(\omega ){\hat{f}}_{r}(\omega )\right\|}_{2}^{2}+{\left\|\hat{f}(\omega )-\left({\hat{u}}_{d}(\omega )+{\hat{f}}_{r}(\omega )\right)+\frac{\hat{\lambda }(\omega )}{2}\right\|}_{2}^{2}\} \end{array}$$

To simplify the above Equation (8), according to Equation (3) and some algebraic manipulations, the mode components ${\hat{u}}_{d}^{n+1}$ at the *n* + 1 iteration can be rewritten by:

(9) $${\hat{u}}_{d}^{n+1}(\omega )=\frac{\hat{f}(\omega )+\alpha^{2}{\left(\omega -\omega_{d}^{n}\right)}^{4}{\hat{u}}_{d}^{n}(\omega )+\frac{\hat{\lambda }(\omega )}{2}}{\left[1+\alpha^{2}{\left(\omega -\omega_{d}^{n}\right)}^{4}][1+2\alpha {\left(\omega -\omega_{d}^{n}\right)}^{2}\right]}$$

To minimize the Equation (11) with respect to $\omega_{d}$, according to some approximate calculations, in the *n* + 1 iteration, the mode center-frequency $\omega_{d}^{n+1}$ can approximately be expressed as:

(10) $$\omega_{d}^{n+1}=\frac{\int_{0}^{\infty }\omega {\left|{\hat{u}}_{d}^{n+1}(\omega )\right|}^{2}d\omega }{\int_{0}^{\infty }{\left|{\hat{u}}_{d}^{n+1}(\omega )\right|}^{2}d\omega }$$

Finally, the dual ascent method is used to update the Lagrangian multiplier λ of ADMM, that is

(11) $${\hat{\lambda }}^{n+1}(\omega )={\hat{\lambda }}^{n}+\tau \left[\hat{f}(\omega )-\left({\hat{u}}_{d}(\omega )+{\hat{f}}_{r}^{n}(\omega )\right)\right]$$

where τ denotes the update parameter which amounts to time-step of the dual ascent. The specific procedure of VME can be found in the original literature [19] and the VME code is available on the Mathworks website.

### 2.2. Parameter Adaptive Variational Mode Extraction

**Figure 1:**
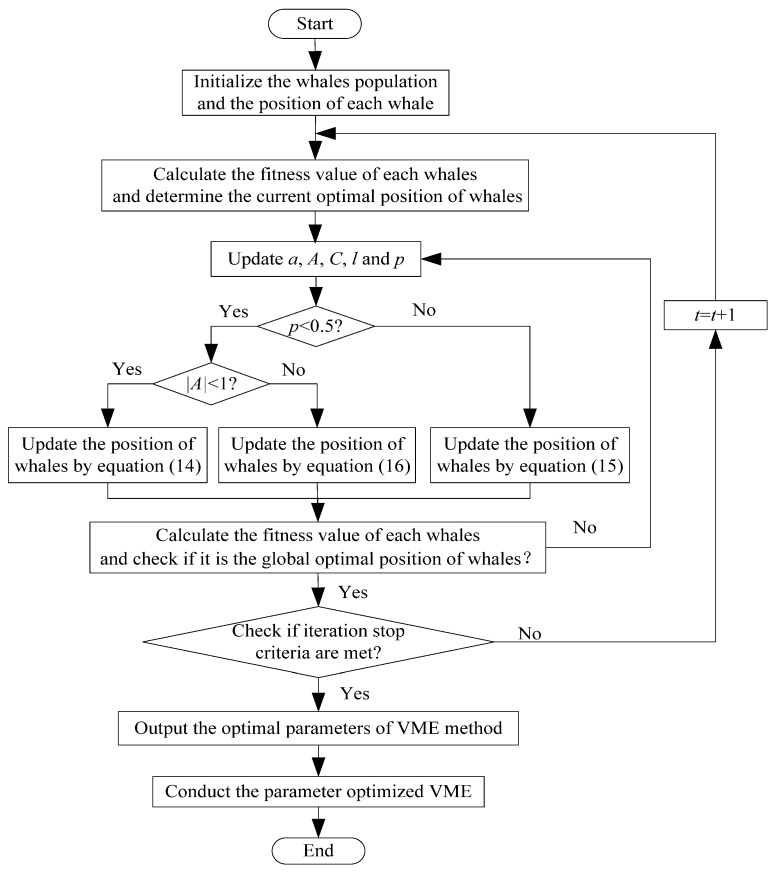
The flowchart of using WOA to optimize the parameters of VME.

When VME is used to process the collected bearing vibration signal, its two important parameters (i.e., penalty factor α and mode center-frequency $\omega_{d}$) need to be artificially selected in advance. Thus, it does not possess adaptive capability. In other words, the parameter setting of VME has a big effect on its feature extraction performance. Due to the penalty factor α controls the compactness of the obtained mode components, so the smaller penalty factor α describes the larger bandwidth of mode components. The closer the predefined mode center-frequency $\omega_{d}$ is to the true center frequency of the desired mode components, the better the feature extraction ability of VME is. Therefore, a suitable method needs to be adopted to automatically select the important parameters of VME. Whale optimization algorithm (WOA) [34] is a recently reported intelligent optimizer, which can mimic bubble-net foraging behavior of humpback whales by applying a bubble-net search mechanism. Compared with particle swarm optimization (PSO), cuckoo search algorithm (CSA), firefly algorithm (FA) and grey wolf optimizer (GWO), WOA has a faster convergence speed, higher convergence accuracy and stronger ability of extremum optimization [35]. Hence, to avoid the problem of empirical selection of the key parameters of VME, a parameter adaptive variational mode extraction (PAVME) is proposed in this paper, where WOA is adopted to automatically determine two key parameters (i.e., penalty factor α and mode center-frequency $\omega_{d}$) of VME, which can improve fault feature extraction ability of VME. Figure 1 shows the flowchart of using WOA to optimize the parameters of VME method. Detailed procedures of parameter optimization in the PAVME are described as follows:

(1) Initialize the population of whales and define the parameters of WOA method. Specifically, set the population size *N* = 50, maximum number of iterations *T* = 200 (i.e., epoch limits). Due to VME involves two key parameters to be optimized, so the position of each whale is expressed by a vector $X^{i}=[\alpha ,f_{d}]$, where α is the penalty factor of VME, $f_{d}$ denotes the initial mode center-frequency of VME and meets $f_{d}=\omega_{d}/2\pi$. The upper and lower bound of the vector $X^{i}$ respectively is set as [200, 10,000] and [$f_{s}/100$, $f_{s}/2$], where $f_{s}$ is the sampling frequency of the raw bearing vibration signal.

(2) Calculate the fitness value of each whales and determine the current optimal position of whales. In this step, inspired by signal-to-noise ratio (SNR) [36] and fault feature ratio (FFR) [37], a new and effective sensitive index hailed as signal characteristic frequency-to-noise ratio (SCFNR) is regarded as the fitness value to guide the parameter optimization process of VME, and the SCFNR index is calculated by

(12) $$\mathrm{SCFNR}(i)=10\log_{10}\frac{\sum_{i=1}^{M}A(f_{ci})}{\sum_{j=1}^{N}A(f_{j})-\sum_{i=1}^{M}A(f_{ci})}$$

where $f_{ci}$ means the *i*-th fault characteristic frequency of Hilbert envelope spectrum of the extracted mode components $u_{d}$, $A(f_{ci}),\;i=1,2,\cdots ,M$ denotes the amplitude of Hilbert envelope spectrum of the original bearing vibration signal at the *i*-th fault characteristic frequency, $A(f_{j}),\;j=1,2,\cdots ,N$ represents the amplitude of Hilbert envelope spectrum of the original bearing vibration signal at the *j*-th frequency *f*, *N* and *M* are the number of all frequencies and fault characteristic frequencies of Hilbert envelope spectrum of the original bearing vibration signal, respectively. The larger SCFNR value represents the better feature extraction ability of VME. That is, parameter optimization process of VME can be understood as the process of maximizing the fitness value (SCFNR). Hence, the objective function of parameter optimization process of VME can be defined as follows:

(13) $$\left\{ \begin{array}{l} \underset{i=(\alpha ,f_{d})}{\arg\max}{\{\mathrm{SCFNR}}_{i}\}\\ s.t.\;\alpha \in [200,\;10000]\;\mathrm{and}\;f_{d}\in [f_{s}/100,\;f_{s}/2] \end{array}\right.$$

where $SCFNR_{i}$ denotes the SCFNR value of the extracted mode components under different combination parameters $i=(\alpha ,f_{d})$, $f_{s}$ represents the sampling frequency of the original bearing vibration signal.

(3) Before reaching the stop condition, update the parameters *a*, *A*, *C*, *l* and *p* under each iteration. If p<0.5, the position updating pattern of the shrinking encircling mechanism of whales is adopted. Otherwise, the position updating pattern of the spiral model of whales is adopted. That is, the probability of selecting the shrinking encircling mechanism or the spiral model to update the position of whales is the same. Concretely, if p<0.5 and |A|<1, update the position of the current whale according to Equation (14). If p≥0.5, update the position of the current whale according to Equation (15). If p<0.5 and |A|≥1, update the position of the current whale according to the randomly prey search mechanism of Equation (16).

(14) $$X(t+1)=X^{*}(t)-A\left|C\cdot X^{*}(t)-X(t)\right|\;\mathrm{if}\;p<0.5\;\mathrm{and}\;\left|A\right|<1$$

(15) $$X(t+1)=\left|C\cdot X^{*}(t)-X(t)\right|\;\cdot e^{bl}\cdot \cos(2\pi l)+X^{*}(t)\;\mathrm{if}\;p\geq 0.5$$

(16) $$\left\{ \begin{array}{l} \begin{array}{cc} X(t+1)=X_{\mathrm{rand}}(t)-A\cdot \left|C\cdot X_{\mathrm{rand}}(t)-X(t)\right| & \;\mathrm{if}\;p<0.5\;\mathrm{and} \end{array}\;\left|A\right|\geq 1\;\\ D=\left|C\cdot X_{\mathrm{rand}}(t)-X(t)\right| \end{array}\right.$$

where *X* is a position vector for all whales, *t* is the time or iteration metrics, X^*^ is the current optimal solution, *A* and *C* represent the coefficient vector and they meets A=2a⋅r−a and C=2⋅r, *a* is a convergence factor that linearly decays from 2 to 0 throughout all iterations, *r* is a random vector between 0 and 1, *b* is a constant value that defines a logarithmic spiral shape in terms of a particular path, *l* is a random value between −1 and 1, *p* is a random value between 0 and 1, which can be used to switch Equations (14) and (15) when updating the position of whales. $X_{\mathrm{rand}}$ represents the position vector for the randomly selected whales in the current iteration, *D* denotes distance of the *i*-th whale to the prey, *A* and *C* represent the coefficient vector.

(4) Calculate the fitness value of each whales and determine the global optimal position of whales. If ${\hat{X}}^{i}$ is better than $X^{i}$, ${\hat{X}}^{i}$ is regarded as the global optimal position of whales. Otherwise, keep $X^{i}$ as the individual optimal position to continue to update.

(5) Check that the stop condition is met. Specifically, determine whether the largest SCFNR value or maximum iteration number is reached. If it reaches the largest SCFNR value or maximum iteration number, output the optimized results (i.e., the optimal parameters of VME). Otherwise, define *t* = *t* + 1, continue to conduct steps (3)–(4) until the stop condition is met.

(6) Use the parameter optimized VME to extract the desired mode components of the collected bearing vibration signal.

**Figure 2:**
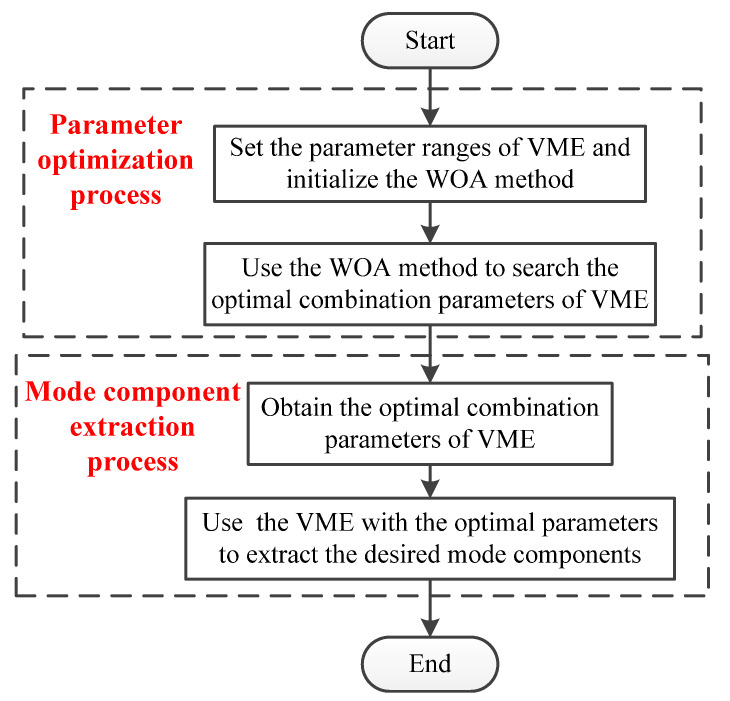
The block diagram of PAVME.

Briefly speaking, the proposed PAVME method mainly consists of two sub-blocks (i.e., parameter optimization process and mode component extraction process). Figure 2 shows the block diagram of PAVME. Therein, the first sub-block is the parameter optimization process based on WOA method, which is aimed at obtaining the optimal combination parameters (i.e., penalty factor α and mode center-frequency $\omega_{d}$) of VME. The second sub-block is mode component extraction process based on VME containing the optimal combination parameters.

### 2.3. Comparison among PAVME, VME, VMD and EMD

**Figure 3:**
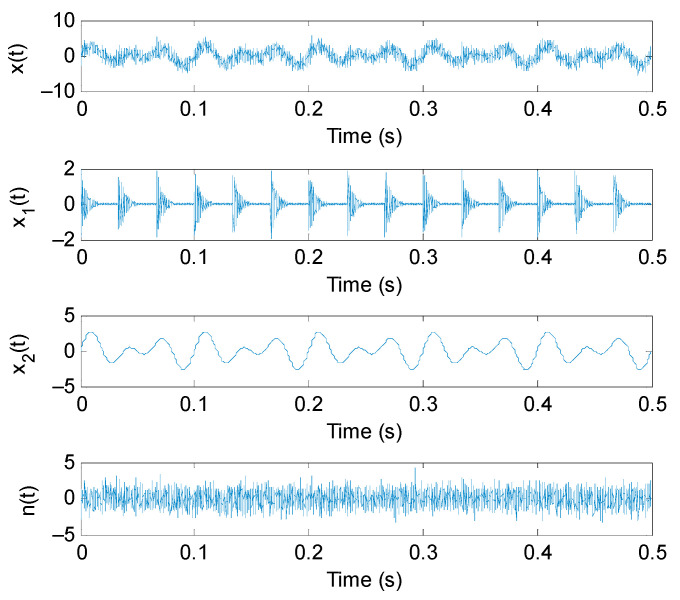
Time domain waveform of simulation signal *x*(*t*) and its corresponding components.

To show the effectiveness of PAVME in extracting periodic impulse features of bearing vibration signal, according to the literature [36], here we established one bearing fault simulation signal *x*(*t*), which is mainly composed of three parts (i.e., *x*_1_(*t*), *x*_2_(*t*) and *n*(*t*)). The specific expression of simulation signal is as follows:

(17) $$\left\{ \begin{array}{l} x(t)=x_{1}(t)+x_{2}(t)+n(t)\\ x_{1}(t)=2\exp(-200t_{0})\sin(4000\pi t),\;t_{0}=\mathrm{mod}(t,1/f_{0})\\ x_{2}(t)=1.3\sin(2\pi f_{2}t)+1.5\sin(2\pi f_{3}t) \end{array}\right.$$

where the first part $x_{1}(t)$ denotes the periodic impulse series related to bearing faults, $f_{o}$ is the bearing fault characteristic frequency and meets $f_{o}$ = 30 Hz. The second part $x_{2}(t)$ represents the harmonic component with the frequency of *f*_2_ = 20 Hz and *f*_3_ = 30 Hz. The third part n(t) represents the Gaussian white noise generated by MATLAB function randn(1,N). The sampling frequency and sampling length of simulation signal *x*(*t*) are set as 8192 Hz and 4096 points, respectively. Figure 3 shows time domain waveform of simulation signal *x*(*t*) and its corresponding components.

**Figure 4:**
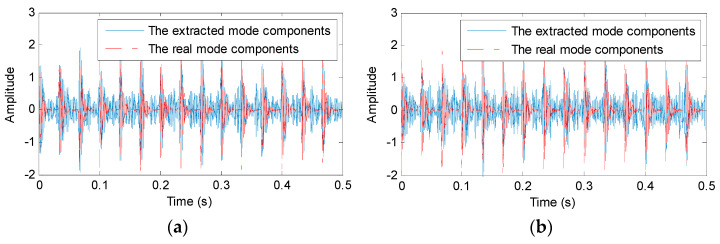

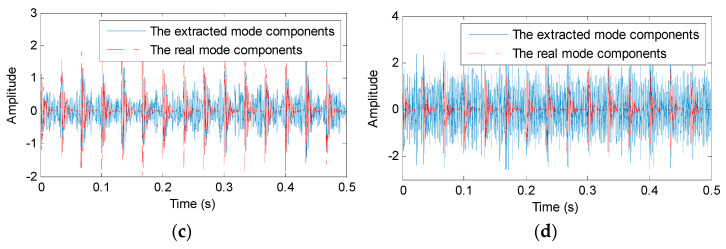
The periodic mode components extracted by different methods: (**a**) PAVME, (**b**) VME, (**c**) VMD and (**d**) EMD.

The proposed PAVME and three standard methods (VME, VMD and EMD) are adopted to process the simulation signal *x*(*t*). In PAVME, the penalty factor α and mode center-frequency $f_{d}$ are automatically selected as 1680 and 2025 Hz by using WOA. In the standard VME, the combination parameters (i.e., penalty factor α and mode center-frequency $f_{d}$) are artificially set as 2000 and 2500 Hz. In VMD, the decomposition mode number *K* and penalty factor α are also automatically selected as 4 and 2270 Hz by using WOA. Figure 4 shows the periodic mode components extracted by different methods (i.e., PAVME, VME, VMD and EMD). Seen from Figure 4, although three methods (PAVME, VME and VMD) can all obtain the periodic impulse features of simulation signal, but their obtained results are different. The periodic mode components extracted by EMD have a big difference with the real mode component $x_{1}(t)$ of the simulation signal. Hence, for a better comparison, fault feature extraction performance of the four methods (PAVME, VME, VMD and EMD) is quantitatively compared by calculating four evaluation indexes (i.e., kurtosis, correlation coefficient, root-mean-square error (RMSE) and running time). Table 1 lists the calculation results. Seen from Table 1, kurtosis and correlation coefficient of the proposed PAVME method is higher than that of other three methods (i.e., VME, VMD and EMD). The RMSE of the PAVME method is less than that of other three methods. This means that the proposed PAVME has better feature extraction performance. However, the running time of VMD is highest, the second is PAVME and the smallest running time is EMD. This because the PAVME and VMD are optimized by WOA, so their computational efficiency is reduced, but it is acceptable for most occasions. The above comparison shows that the PAVME method is effective in bearing fault feature extraction.

## 3. Multiscale Envelope Dispersion Entropy

### 3.1. MEDE

**Figure 5:**
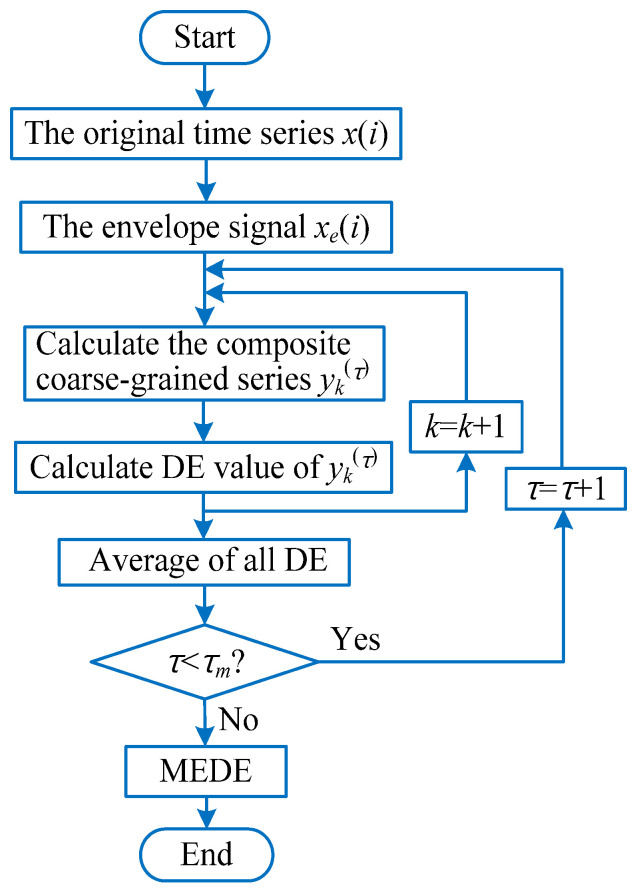
Flowchart of the MEDE method.

On the one hand, envelope demodulation analysis of bearing vibration signals is an effective method in extracting bearing fault feature information. The extracted envelope signal can nicely reflect the characteristics of periodic impulse related to bearing local faults. On the other hand, entropy has been proved to be an effective method to describe the complexity and uncertainty of bearing vibration signal. Some studies [32,38] have shown that multiscale dispersion entropy (MDE) has the superior performance for measuring the complexity of a signal than MPE and MSE. MDE has a faster calculation efficiency. Hence, this paper proposes a new complexity evaluation method named multiscale envelope dispersion entropy (MEDE) by integrating the advantages of envelope demodulation analysis and MDE. Figure 5 shows the flowchart of the MEDE method, where $\tau_{m}$ means the defined largest scale factor. For a given time series {x(i),i=1,2,⋯,N}, the specific steps of MEDE are summarized as follows:

(1) Conduct envelope demodulation analysis. Specifically, use the Equation (18) to calculate the envelope signal of the original time series *x*(*i*).

(18) $$x_{e}(i)=\left|x(i)+j\cdot \mathrm{Hilbert}[x(i)]\right|$$

where *x*(*i*) represents the given original signal, $x_{e}(i)$ denotes the envelope signal of *x*(*i*), Hilbert[·] represents the Hilbert transform operator.

(2) According to the Equation (19), calculate the composite coarse-grained time series $y_{k}^{\left(\tau \right)}=\{y_{k,j_{1}}^{\left(\tau \right)},y_{k,j_{2}}^{\left(\tau \right)},\cdots ,y_{k,j_{\tau }}^{\left(\tau \right)}\}$ of the envelope signal $x_{e}(i)$. Specifically, when the scale factor τ = 1, the obtained composite coarse-grained time series $y_{k}^{\left(1\right)}$, amounts to the original envelope signal $x_{e}(i)$. When the scale factor τ = 2, we will obtain two composite coarse-grained time series $y_{k}^{\left(1\right)}$ and $y_{k}^{\left(2\right)}$.

(19) $$y_{k,j}^{\left(\tau \right)}=\frac{1}{\tau }\sum_{i=(j-1)\tau +k}^{j\tau +k-1}x_{e}(i),\;1\leq j\leq \left\lfloor \frac{N}{\tau }\right\rfloor ,\;1\leq k\leq \tau$$

(3) Calculate the dispersion entropy value of each composite coarse-grained time series $y_{k}^{\left(\tau \right)}(k=1,2,\cdots ,\tau )$ at the scale factor τ. Moreover, the average operation of all dispersion entropy is conducted to obtain the final MEDE results.

(20) $$\mathrm{MEDE}(x,m,c,d,\tau )=\frac{1}{\tau }\sum_{k=1}^{\tau }DE(y_{k}^{\left(\tau \right)}m,c,d)$$

where *m* denotes the embedding dimension, c means the number of classes, *d* is the time delay, τ represents the scale factor and DE(·) denotes the operator of dispersion entropy. According to reference [33], without loss of generality, the embedding dimension *m* is usually set as 3, the number of classes *c* is usually set as 5 or 6, the time delay *d* is usually set as 1, the largest scale factor $\tau_{m}$ is usually set as 20, which are enough for the practical bearing vibration signal analysis and complexity evaluation.

### 3.2. Comparison among MEDE, MDE, MPE and MSE

**Figure 6:**
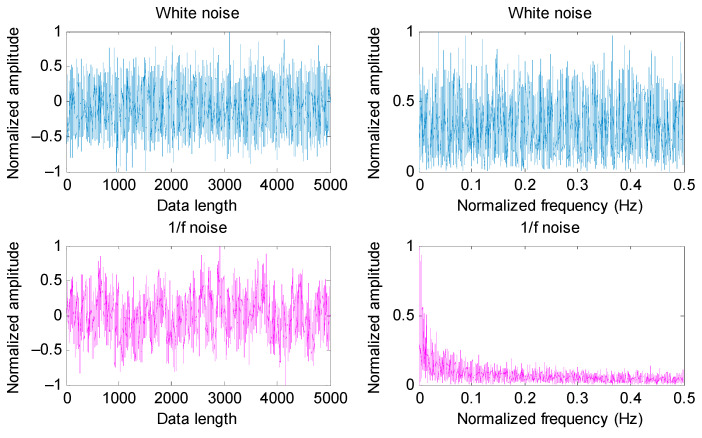
Time domain waveform and amplitude spectrum of two noise signals (i.e., white noise and 1/f noise).

**Figure 7:**
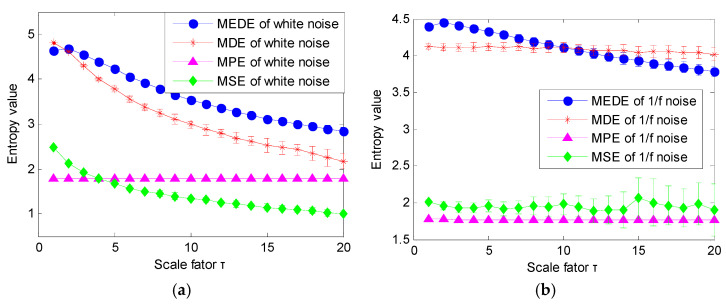
Entropy value obtained by different methods for two noise signals: (**a**) white noise and (**b**) 1/f noise.

To show the effectiveness of MEDE in evaluating the complexity and irregularity of a time series, MEDE of two noise signals (i.e., white noise and 1/f noise) are calculated. For a convenient comparison, three common entropies (i.e., MDE, MPE and MSE) of two noise signals (i.e., white noise and 1/f noise) are calculated to measure the complexity of the time series. Also, to compare the accuracy of complexity measures of different entropies, 20 groups of white noise and 1/f noise are generated randomly. Figure 6 shows time domain waveform and amplitude spectrum of a group of white noise and 1/f noise. Figure 7a,b plot the error bar of different entropies (i.e., MEDE, MDE, MPE and MSE) of white noise and 1/f noise, respectively. Seen from Figure 7a, as the scale factor τ increases, mean value curve of three entropies (i.e., MEDE, MDE and MSE) of white noise have a downward trend, whereas the mean value curve of MPE of white noise basically remains unchanged. That is, the sensitivity of MEDE, MDE and MSE in detecting complexity of white noise is better than MPE. As shown in Figure 7a, standard deviation of MEDE of white noise at each scale factor τ is obviously smaller than MDE. That indicates that MEDE has a better accuracy in complexity measures of white noise than MDE. Seen from Figure 7b, as the scale factor τ increases, the mean value curve of three entropies (i.e., MDE, MPE and MSE) of 1/f noise is relatively stable, whereas mean value curve of MEDE of 1/f noise decreases gradually, which means that MEDE is more sensitive for uncertainty estimation of 1/f noise than other three entropies (i.e., MDE, MPE and MSE). Moreover, in Figure 7b, standard deviation of MEDE of 1/f noise at each scale is less than that of MDE and MSE. This further validates that MEDE can provide an accurate complexity estimation for 1/f noise. That is, MEDE is effective in complexity measurement and feature extraction of nonstationary signals.

## 4. Proposed Fault Diagnosis Method

**Figure 8:**
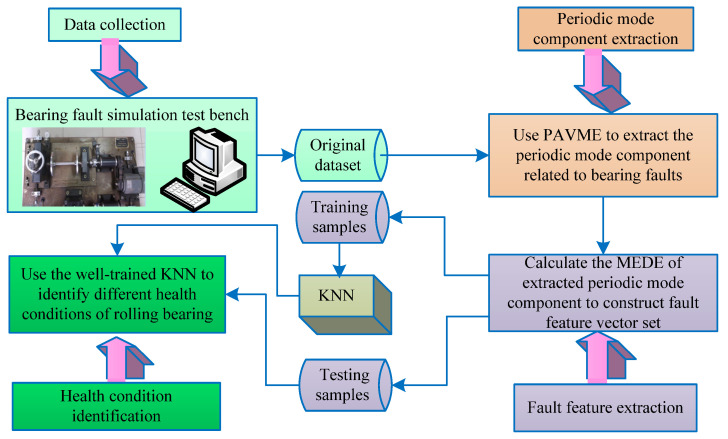
Flowchart of the proposed method for bearing fault identification.

To effectively extract feature information associated with bearing local fault and automatically realize the identification of bearing health status, this paper proposes a new bearing fault diagnosis method based on PAVME and MEDE, which mainly consists of four aspects (i.e., vibration data collection, periodic mode component extraction, fault feature extraction and health condition identification). Figure 8 shows the flowchart of the proposed method. The specific steps of the proposed method are summarized as follows:

Step 1: vibration data collection. Collect the original bearing vibration signal by installing the accelerometer on the bearing fault simulation test bench.
Step 2: speriodic mode component extraction. Use the PAVME method to extract the periodic mode component related to bearing faults, where the WOA method is adopted to automatically determine the optimal combination parameters of VME.
Step 3: fault feature extraction. Calculate the MEDE of the extracted periodic mode component to construct multiscale fault feature vector set.
Step 4: health condition identification. In view of k-nearest neighbor (KNN) has the less parametric influence and faster computing speed than support vector machine (SVM) and artificial neural network (ANN), so the KNN classifier is selected in this step. Concretely, the constructed multiscale fault feature vector set in step 3 is randomly divided into the training samples and testing samples, where the training samples are adopted to train the KNN model and the testing samples is fed into the well-trained KNN model to automatically identify different health conditions of rolling bearing. Note that, in the KNN classifier, based on the previous studies [39], the Euclidean distance is adopted and the number of nearest neighbors of KNN is set as 3. Of course, in the KNN classifier the Mahalanobis distance, Chebyshev distance and the larger neighbor number can be also adopted, but too large neighbor number tends to cause the low identification accuracy. Generally speaking, the number of nearest neighbors should be less than the square root of the training sample number.

## 5. Experimental Verification

### 5.1. Case 1: Bearing Data from Laboratory

#### 5.1.1. Experimental Equipment Description and Data Collection

**Figure 9:**
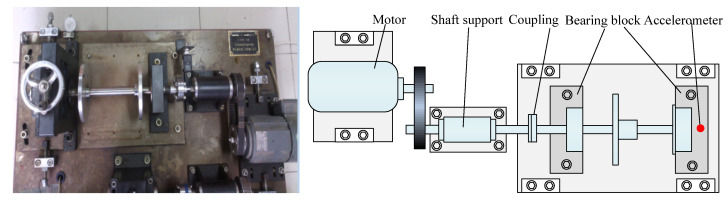
Picture and schematic drawing of experimental equipment.

**Figure 10:**
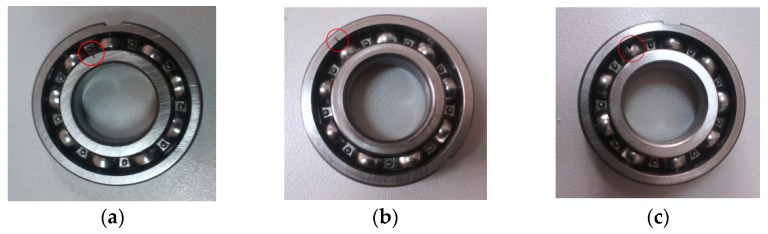
Pictures of the faulty bearings: (**a**) IRF, (**b**) ORF and (**c**) BF.

**Figure 11:**
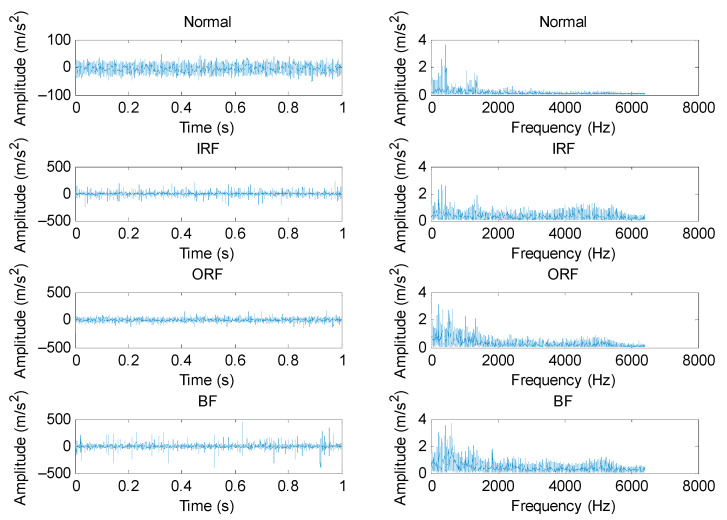
Time domain waveform and amplitude spectrum of different bearing vibration signal.

To validate the effectiveness of the proposed method, different bearing vibration signals were collected on a bearing fault simulation test rig located in North China Electric Power University (NCEPU). Figure 9 shows the photo and structural schematic diagram of the bearing fault simulator, which mainly consists of a driving motor, transmission belt, shaft support, coupling and bearing block. Within the experiment, three kinds of faults (i.e., inner race fault (IRF), outer race fault (ORF) and ball fault (BF)) were manufactured on normal bearings by electrospark wire-electrode cutting. The size of the outer and inner race faults was set as 0.008 inches in width and 0.059 inches in depth. Figure 10 gives the pictures of three faulty bearings. In the process of experiment, the spindle speed was stable at 1470 r/min and the signal sampling frequency is set as 12.8 kHz. We used a PCB accelerometer mounted on the vertical direction of the testing bearing to collect bearing vibration data under four health conditions (i.e., normal, IRF, ORF and BF). The types of the testing bearing is LYC6205E and Table 2 lists the specification of rolling bearings. According to the spindle speed and the size parameter of bearing, different bearing fault characteristic frequencies (e.g., inner race, outer race, ball and cage) are derived in Table 3. One hundred data samples of each health condition were obtained by using a sliding window with 80% overlap (i.e., 10,240 points) to conduct the data interception along the original bearing vibration signal, and each sample has 12,800 points (i.e., the window size is 12,800 data points), where 50 samples are randomly selected as the training samples and the remainder 50 samples are regarded as the testing samples. Table 4 lists the detailed description of bearing datasets. As shown in Table 4, there are 400 samples in total. That is, a four-class identification problem needs to be solved in this experiment. Figure 11 shows the time domain waveform and amplitude spectrum of different bearing vibration signals. It can be seen from Figure 11 that the waveform and amplitude spectrum of the bearing fault signal have a certain similarity, which indicates that it is not easy to directly judge the bearing fault type by observing the waveform and amplitude spectrum. In other words, there is an urgent need for exploring an effective method to identify bearing fault types.

#### 5.1.2. Periodic Mode Component Extraction Based on PAVME

**Figure 12:**
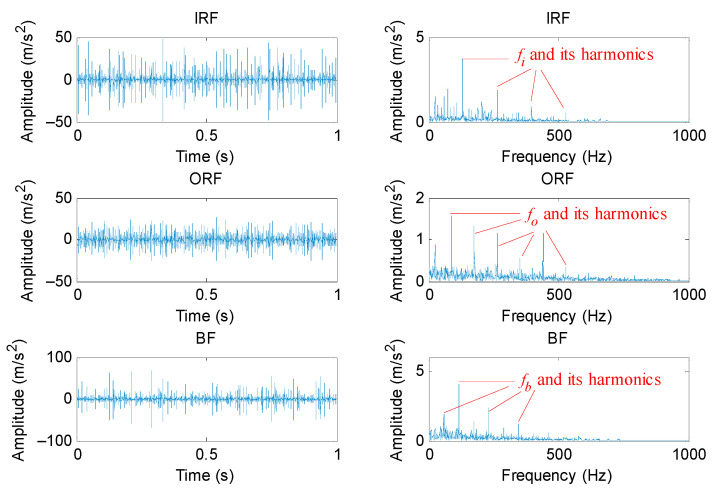
Time domain waveform and envelope spectrum of periodic mode components obtained by PAVME for different bearing fault signals.

**Figure 13:**
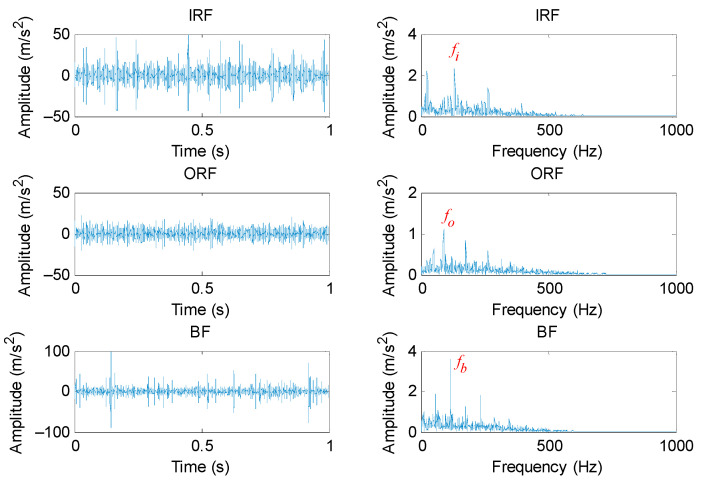
Time domain waveform and envelope spectrum of periodic mode components obtained by VME for different bearing fault signals.

**Figure 14:**
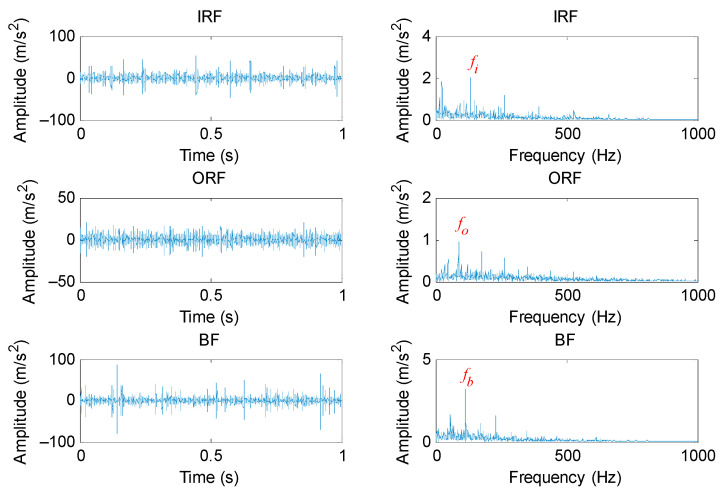
Time domain waveform and envelope spectrum of periodic mode components obtained by VMD for different bearing fault signals.

**Figure 15:**
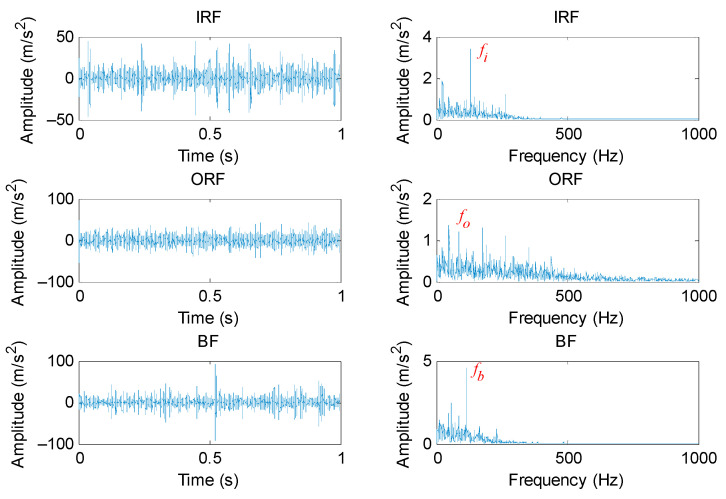
Time domain waveform and envelope spectrum of periodic mode components obtained by EMD for different bearing fault signals.

According to the flowchart of the proposed method, the PAVME was firstly applied to preprocess the original bearing vibration signal, where its two key parameters (i.e., the penalty factor and mode center-frequency) were automatically determined by WOA. It should be noted that normal bearing signals were not processed by PAVME. Table 5 lists the optimal combination parameters of VME for different bearing fault signals. Figure 12 shows the time domain waveform and envelope spectrum of periodic mode components obtained by PAVME for different bearing fault signals. As shown in the envelope spectrum of Figure 12, when bearing fault signals were analyzed by PAVME, three kinds of bearing fault feature frequencies (i.e., inner race fault feature frequencies *f_i_*, outer race fault feature frequencies *f_o_* and ball fault feature frequencies *f_b_*) and their harmonics could be clearly extracted, which indicates that the proposed PAVME is effective in extracting periodic mode components related to bearing faults. For a comparison, three similar methods (i.e., VME, VMD and EMD) were also used to analyze the collected bearing fault signal. It needs to be emphasized that periodic mode component with maximum kurtosis was selected for fault feature extraction in the decomposition results of VMD and EMD. In VME and VMD, the penalty factor was set as the same as PAVME. Meanwhile, for VME, the mode center-frequency was always set as *f_d_* = 2500 Hz, whereas the decomposition mode number was always set as *K* = 6 for VMD. Figure 13, Figure 14 and Figure 15 show the time domain waveform and envelope spectrum of periodic mode components obtained by three methods (i.e., VME, VMD and EMD) for different bearing fault signals, respectively. As shown by Figure 13, Figure 14 and Figure 15, although three kinds of bearing fault feature frequencies (i.e., inner race fault feature frequencies *f_i_*, outer race fault feature frequencies *f_o_* and ball fault feature frequencies *f_b_*) and their harmonics could be found, their amplitudes were lower than that of Figure 12. That is, compared with three similar methods (i.e., VME, VMD and EMD), PAVME had a better extraction ability of periodic mode component and is more helpful for the subsequent bearing fault feature extraction. Hence, the above comparison proves the advantages of PAVME in processing bearing fault signals.

#### 5.1.3. Results and Comparisons of Bearing Fault Identification

**Figure 16:**
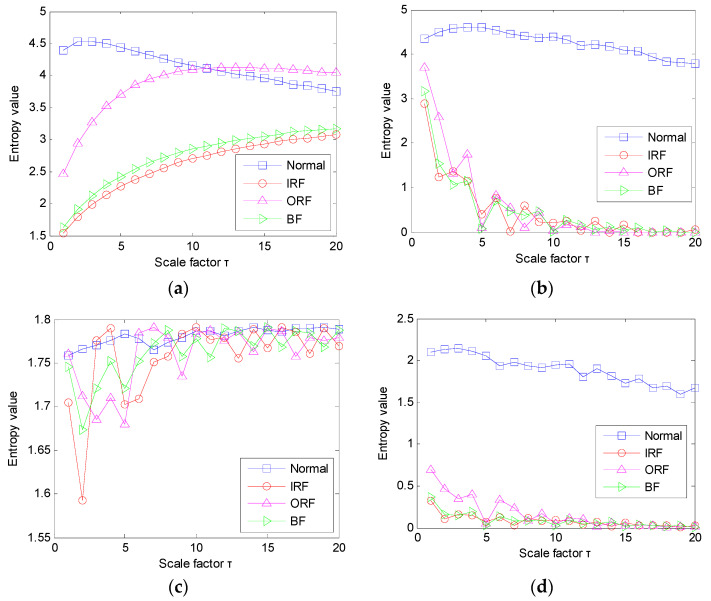
Entropy value obtained by four combination methods for different bearing vibration data: (**a**) PAVME and MEDE, (**b**) PAVME and MDE, (**c**) PAVME and MPE, (**d**) PAVME and MSE.

In the proposed method, after conducting PAVME, the MEDE of the obtained periodic mode component is calculated to extract bearing fault feature information. For a fair comparison, the other three methods (i.e., MDE, MPE and MSE) were also adopted for fault feature extraction. In these entropy methods, their main parameters were set to be the same. Specifically, in MEDE and MDE, the embedding dimension *m* = 3, the number of classes *c* = 5, the time delay *d* = 1, the largest scale factor $\tau_{m}$ = 20. In the MPE method, the embedding dimension *m* = 3, the time delay *d* = 1, the largest scale factor $\tau_{m}$ = 20. In the MSE method, the embedding dimension *m* = 3, the time delay *d* = 1, the tolerance *r* = 0.15 × *σ*, the largest scale factor $\tau_{m}$ = 20, where *σ* represents the standard deviation of the signal. Figure 16a–d) show entropy values obtained by combining PAVME and four entropies (i.e., MEDE, MDE, MPE and MSE) for different bearing vibration signals. Apparently, in Figure 16, the entropy value obtained using PAVME and MEDE has a good degree of differentiation, whereas the entropy value obtained using other combination methods (i.e., PAVME and MDE, PAVME and MPE, PAVME and MSE) has an obvious overlap, particularly for the entropy value of bearing fault signal. This verifies the effectiveness of MEDE in bearing fault feature extraction to a certain extent.

**Figure 17:**
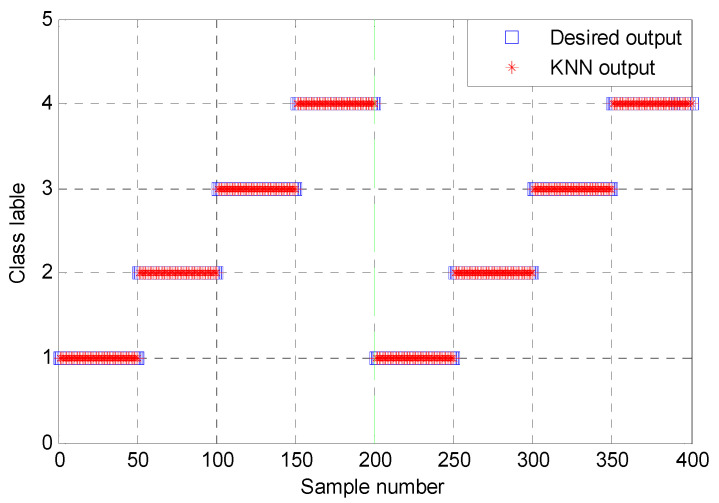
Identification results of the first trial of the proposed method in case 1.

**Figure 18:**
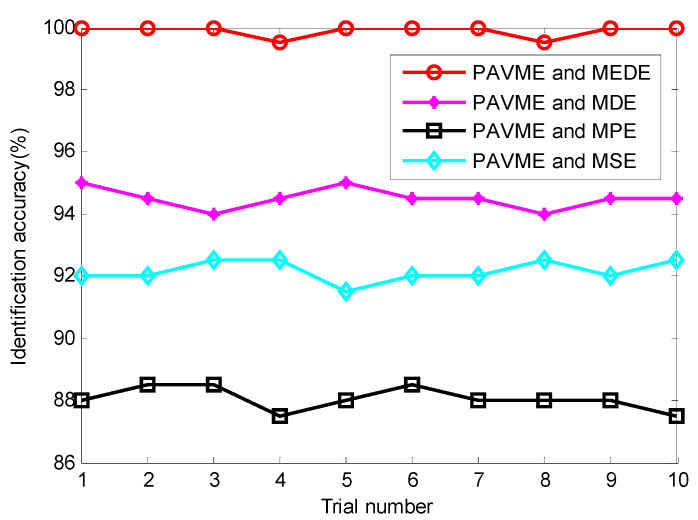
Identification accuracy obtained by different methods for 10 trials in case 1.

**Figure 19:**
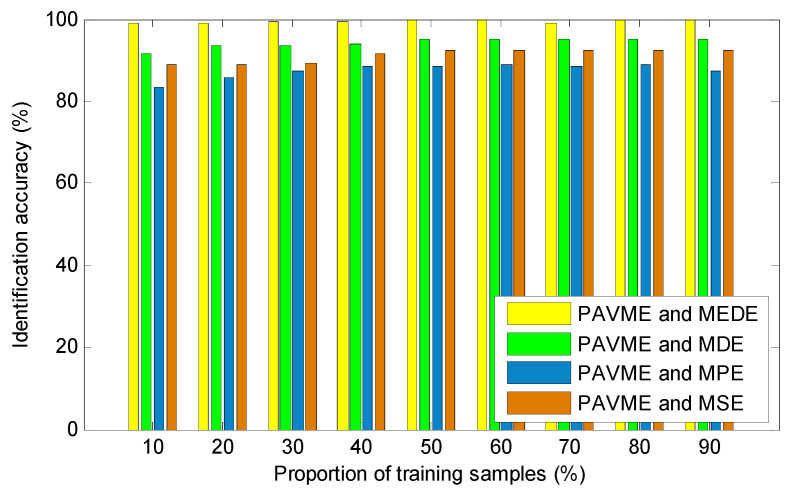
Identification accuracy obtained by combining PAVME and different entropies under different proportion of training samples.

According to the proposed method, finally, the above extracted bearing fault feature information is input into KNN classifier for identifying bearing fault types. Figure 17 shows the identification results of the proposed method in the first trial. Seen from Figure 17, the proposed method can obtain a high identification accuracy of 100%, which indicates that all data samples can be correctly identified. To avoid the contingency of recognition results of the algorithm, four combination methods (i.e., PAVME and MEDE, PAVME and MDE, PAVME and MPE, PAVME and MSE) are conducted five trials to compare their recognition results. Figure 18 shows the identification accuracy of different methods in the five trials and Table 6 lists the detailed diagnosis results of different combination methods, including maximum, minimum, mean and standard deviation of identification accuracy. As shown Figure 18 and Table 6, the method of combining PAVME and MEDE can achieve the average accuracy of 99.90%, which is apparently higher than that of other three combination methods (i.e., PAVME and MDE, PAVME and MPE, PAVME and MSE), which are 94.50%, 88.05% and 92.15%, respectively. That is, the classification accuracy of the proposed method was the highest. The standard deviation of the proposed method (i.e., PAVME and MEDE) is 0.2108, which is obviously lower than that of other three combination methods (i.e., PAVME and MDE, PAVME and MPE, PAVME and MSE), which are 0.3333, 0.3689 and 0.3375, respectively. This indicates that the identification result of the proposed method had better stability. In other words, when PAVME is combined with different entropies (i.e., MEDE, MDE, MPE and MSE) to identify bearing fault patterns, the superiority of MEDE used in the proposed method is confirmed by the above comparative analysis. To further investigate the influence of the number of training samples on the recognition performance of the proposed method, for different training sample ratio (i.e., 10%, 20%, 30%, 40%, 50%, 60%, 70%, 80%, 90%), that is, when the number of training samples was respectively set as 40, 80, 120, 160, 200, 240, 280, 320 and 360, the identification results of four combination methods (i.e., PAVME and MEDE, PAVME and MDE, PAVME and MPE, PAVME and MSE) were calculated. Note that the training samples were randomly selected from the collected whole sample set. Also, each combination of methods had 10 trials to avoid volatility in the identification results. Figure 19 shows the average identification accuracy of four combination methods under different proportion of training samples. Seen from Figure 19, the identification accuracy of the proposed method was still bigger than that of other combination methods, even if the proportion of training samples were set as 10%. In addition, as the proportion of training samples increased, the identification accuracy of various methods had a slow upward trend. Theoretically, the more training samples there are, the better the training of the classification model is, the higher the obtained identification accuracy is. However, more training samples represents more training time and lower computational efficiency. Therefore, to strike a balance between the identification accuracy and training time, the proportion of training samples was set as 50% in this paper.

**Figure 20:**
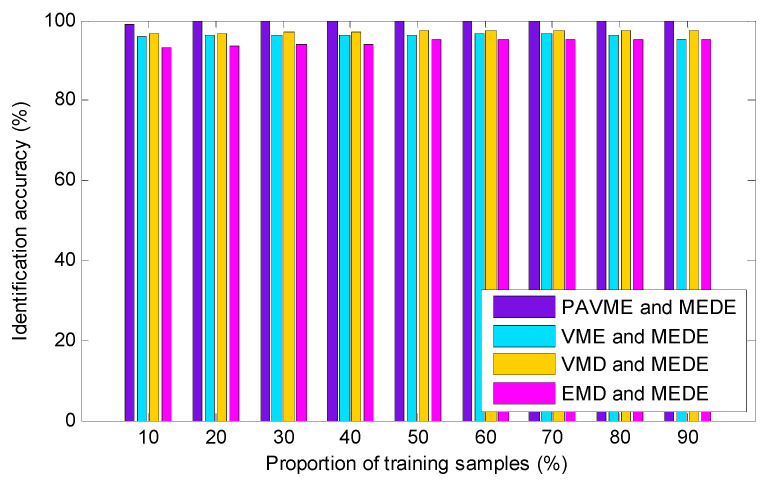
Identification accuracy obtained by combining different signal processing methods and MEDE for different proportions of training samples.

To show the effectiveness and superiority of PAVME used in the proposed method, we calculated the identification results of combining four signal processing methods (i.e., PAVME, VME, VMD and EMD) and MEDE. Similarly, 10 trials were executed for each method. Table 7 gives the detailed identification results of various methods, including maximum, minimum, mean and standard deviation of identification accuracy. It can be found in Table 7 that average identification accuracy of the four combination methods (i.e., PAVME and MEDE, VME and MEDE, VMD and MEDE, EMD and MEDE) was respectively 99.90%, 96.85%, 97.85% and 95.25%, where average accuracy of the proposed method was highest and average accuracy of the fourth combination method (i.e., EMD and MEDE) was the smallest. From a standard deviation point of view, the proposed method had the smallest standard deviation (0.2108), which means that the proposed method had not only a good recognition performance but also good stability in analyzing bearing fault data. That is, this indirectly proved that the PAVME is better than the other three similar methods (i.e., VME, VMD and EMD) in processing bearing fault signals. In like manner, to analyze the identification ability of the proposed method under different number of training samples, we calculated the identification accuracy of four combination methods (i.e., PAVME and MEDE, VME and MEDE, VMD and MEDE, EMD and MEDE) under different proportions of training sample, and 10 trials were conducted for each method. Figure 20 plots the identification results of various combination methods under different proportion of training samples. As shown in Figure 20, although the number of training samples gradually increased, average identification accuracy of the proposed method was still greater than that of other three combination methods (i.e., VME and MEDE, VMD and MEDE, EMD and MEDE). It is worth mentioning that accuracy of each combination method was greater than 95.00%, which indicates that all four combination methods can be applied in the identification of actual bearing fault types if the training samples are sufficient. Nevertheless, a lot of training samples will lead to a lot of calculations, so this paper adopts 50% of training samples to extract bearing fault feature information and finish bearing health condition identification, which can ensure a compromise between accuracy and training time.

**Figure 21:**
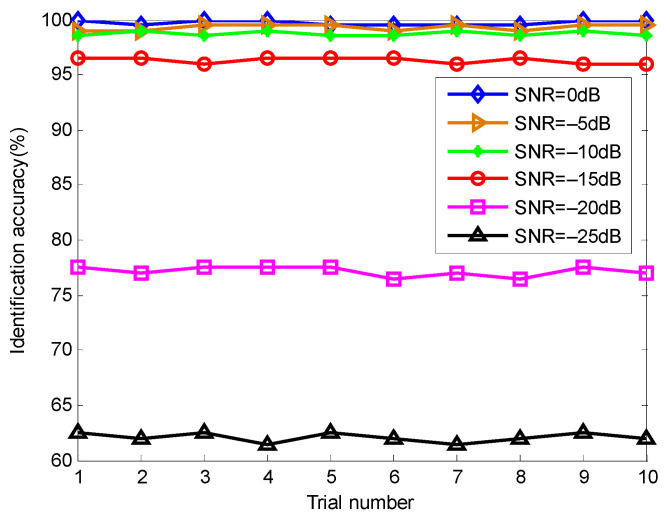
Identification accuracy of the proposed method at different SNR conditions in case 1.

In order to evaluate the influence of Gaussian white noise on the proposed method, according to the literature [40], we added different levels of noises into the original bearing data and calculated the identification results of the proposed method at different noise levels (i.e., SNR = 0, −5, −10, −15, −20 and −25 dB), as shown in Figure 21. Seen from Figure 21, as the SNR decreases, the identification accuracy of the proposed method has a downward trend. However, when Gaussian white noise with SNR = −15 dB was added into the collected original bearing vibration signal, the proposed method could still achieve identification accuracy of more than 95%, which indicates that the proposed method has good robustness in identifying bearing fault patterns.

### 5.2. Case 2: Bearing Data from CWRU

#### 5.2.1. Experimental Equipment Description and Data Collection

**Figure 22:**
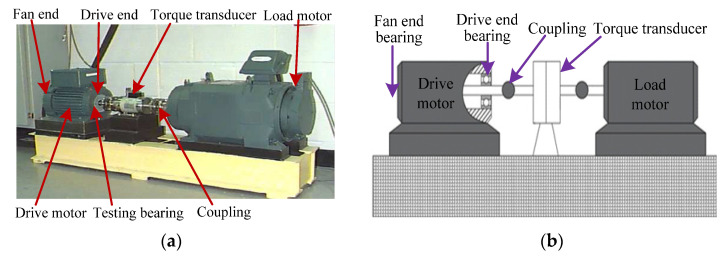
(**a**) The experimental equipment and (**b**) its corresponding structure diagram.

**Figure 23:**
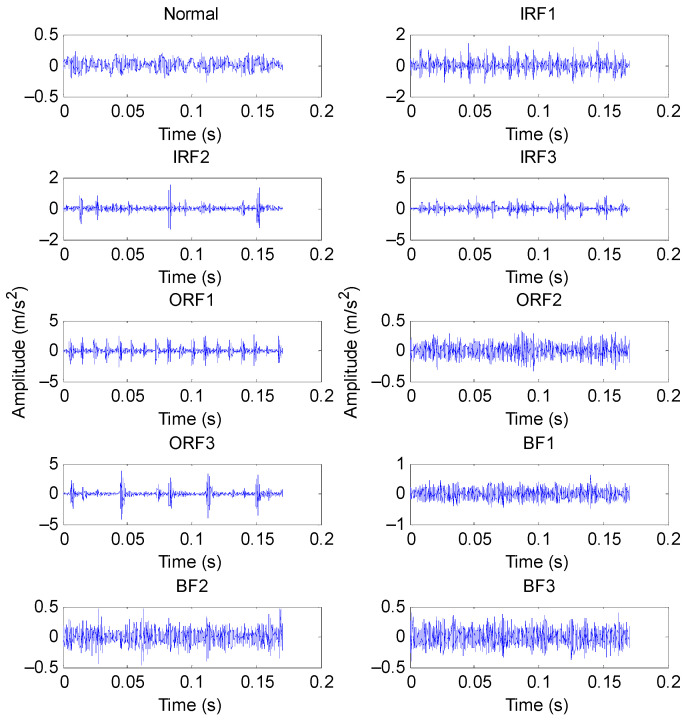
Time domain waveform of bearing vibration data under different health conditions.

Bearing vibration data from Case Western Reserve University (CWRU) were adopted to prove the effectiveness of our proposed approach. Experimental equipment consisted of driving motor, testing bearing, torque transducer and load motor. Figure 22 shows the photos of the experimental equipment and its corresponding structure. Size parameters of the testing bearing are presented in Table 8. In the experiment process, three single-point faults (i.e., inner race fault (IRF), outer race fault (ORF), ball fault (BF)) were manufactured on normal bearings by using electric sparks. One accelerometer with sampling frequency of 12 kHz was mounted on the bearing block of the drive end of the driving motor to collect bearing vibration data. Bearing vibration data of four health conditions (i.e., Normal, IRF, ORF and BF) were collected at the rotating speed of 1797, 1772, 1750 and 1730 r/min, respectively. Specifically, in this example, normal bearing data under 1797 r/min, IRF data of different fault sizes under 1772 r/min, ORF data of different fault sizes under 1750 r/min, and BF data of different fault sizes under 1730 r/min were adopted, which indicates that the experimental equipment operated at variable speed. There were 10 bearing health conditions in total. Fifty samples of each bearing health conditions were obtained via a nonoverlapping sliding window with the length of 2048 points. That is, each sample had 2048 points. Twenty-five samples of each bearing health conditions are randomly selected as the training set and the remainder 25 data samples are regarded as the testing set. That is, the ratio of training samples to testing samples is 1:1. Table 9 lists the detailed description of bearing vibration data used in this case. Figure 23 plots the time domain waveform of bearing vibration data under different health conditions. Obviously, due to the presence of signal interference and noises, it is very difficult to identify the bearing fault category and severity by directly observing the time domain waveform.

#### 5.2.2. Comparison and Analysis

**Figure 24:**
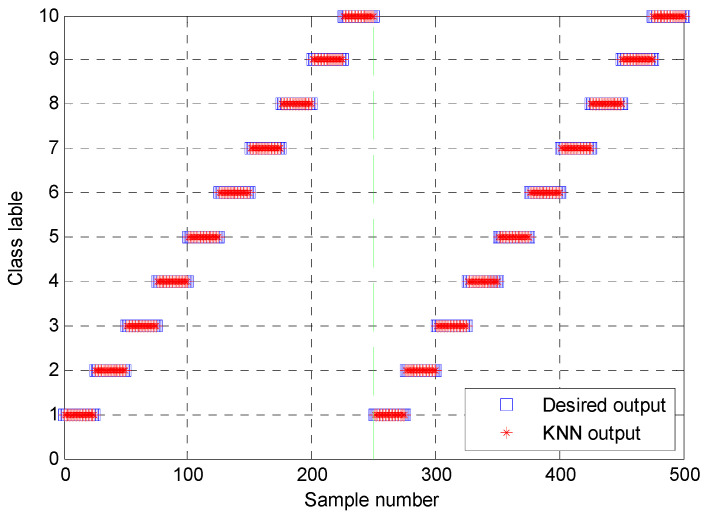
Identification results of the first trial of the proposed method in case 2.

**Figure 25:**
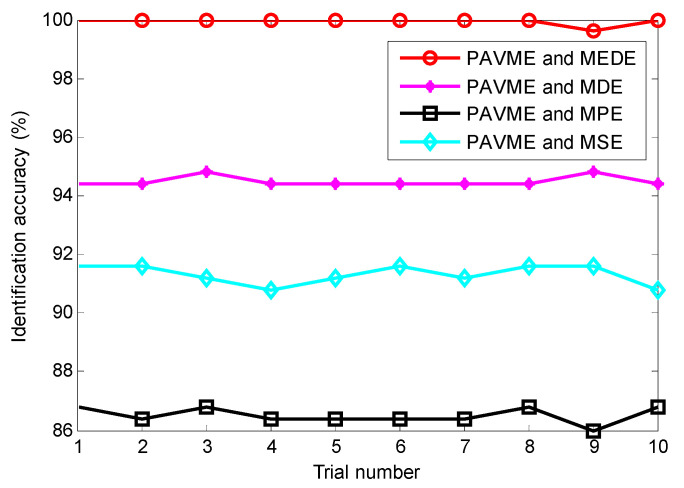
Identification accuracy obtained by different methods for 10 trials in case 2.

The proposed method was used to analyze bearing vibration data under the variable speed and variable fault sizes from CWRU. The optimal combination parameters of PAVME are listed in Table 10. In the MEDE, the embedding dimension *m* = 3, the number of classes *c* = 5, the time delay *d* = 1, the largest scale factor $\tau_{m}$ = 20. Due to the space limitation, here the separate analysis results of PAVME or MEDE were not plotted. Figure 24 shows the direct recognition result of the first trial of the proposed method. As seen in Figure 24, the proposed method can obtain identification accuracy of 100% (250/250) for the training set or testing set. To evaluate the identification performance of the proposed method more reliably, a comparison among different methods (i.e., PAVME and MEDE, PAVME and MDE, PAVME and MPE, PAVME and MSE) was conducted and each method was operated 10 times to objectively evaluate their diagnostic results. The MDE, MPE and MSE had the same parameter setting as case 1. Figure 25 plots the identification results of 10 trials of different methods and Table 11 lists the detailed diagnosis results of different combination methods. It can be found from Figure 25 and Table 11 that average accuracy of the proposed method (i.e., PAVME and MEDE) was 99.96%, which is significantly higher than that of the other three methods (i.e., PAVME and MDE, PAVME and MPE, PAVME and MSE). Moreover, the standard deviation of the proposed method was 0.1265, which is smaller than that other three methods. That is, compared with the above-mentioned comparison methods, the proposed method had better ability and stability in identifying bearing fault categories and fault sizes. Meanwhile, the effectiveness and necessity of MEDE used in the proposed method were verified by this comparison.

To further show the effectiveness of the proposed method and explain the necessity of PAVME in the proposed method, the same bearing vibration data were analyzed by the combination method of different signal processing techniques (i.e., PAVME, VME, VMD and EMD) and MEDE and 10 trials of each method were conducted. Detailed diagnosis results are given in Table 12. As seen in Table 12, the average recognition accuracy (99.96%) of the proposed method was still higher than that of other combined methods, which indirectly indicates that PAVME has a superiority in improving fault identification accuracy. The standard deviation (0.1265) of the proposed method was also less than that of the other combined methods, which means that the proposed method has a good stability. This shows that PAVME used in the proposed method was effective and necessary in bearing fault identification under variable conditions.

**Figure 26:**
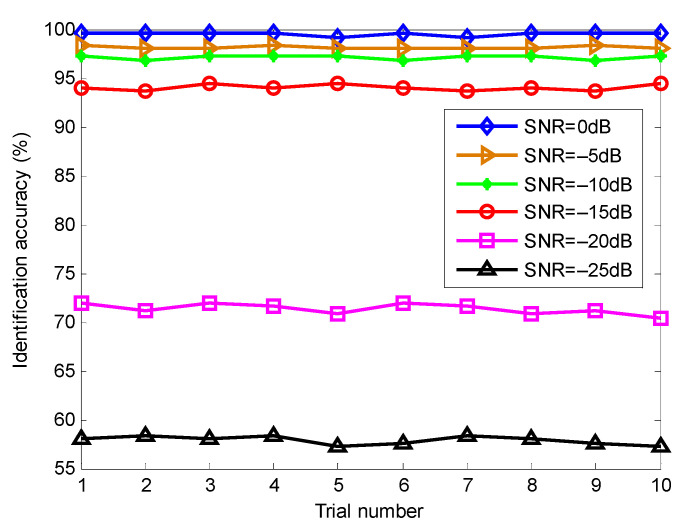
Identification accuracy of the proposed method at different SNR conditions in case 2.

To further consolidate the identification results and evaluate the robustness of the proposed method, the identification accuracy of the proposed method at different noise levels (i.e., SNR = 0, −5, −10, −15, −20 and −25 dB) was calculated and the detailed diagnosis results are plotted in Figure 26. It can be clearly observed from Figure 26 that the recognition performance of the proposed method decreases with the increase of the added noises. Nevertheless, the proposed method can still obtain identification accuracy of 90% above, even if SNR = −15 dB. Therefore, according to the relationship between identification accuracy and SNR, it can also be concluded that the proposed method has a good robustness in identifying bearing faults under variable working conditions.

### 5.3. Further Discussion

It is a fact that the reliability verification of the proposed method is very important for its future real applications. In this paper, from the perspective of the robustness of the algorithm and the comparison of different methods, the effectiveness and superiority of the proposed method are demonstrated by using the above experimental cases under constant and variable speed. However, the proposed method also has some limitations. Specifically, it can be summarized into three aspects:

(1) In the periodic mode component extraction stage of the proposed method, due to the parameter optimizer (WOA) is adopted in PAVME, so it increases the elapsed time of mode component extraction. That is, one limitation of the proposed method can be regarded as the computational speed problem. To address this problem, in future work, some sensitive sparsity indicators (e.g., harmonic-to-noise ratio, kurtosis, L2/L1 norm, Hoyer measure and Gini index) will be adopted to replace the complicated parameter optimizer to automatically select the important parameters of VME. Similar to some traditional optimization algorithms (e.g., particle swarm optimization (PSO), genetic algorithm (GA) and gravitational search algorithm (GSA)), when WOA is used to solve complex optimization problems, it also is affected by the local optimum problem. Therefore, to solve this problem, in the original WOA, the stochastic mechanism or restart strategy will be adopted in our future work.
(2) In the fault feature extraction stage of the proposed method, the performance of MEDE is easily affected by its parameter settings. In this paper, some empirical parameters of MEDE were set to extract bearing fault feature information. Although these empirical parameters have been shown to be effective in bearing fault feature extraction, the prior knowledge is particularly required, so it is not suitable for ordinary technicians with no experience. To address this problem, in future work, some assisted indicators (e.g., Euclidean distance, Mahalanobis distance and Chebyshev distance) could be introduced to automatically select the key parameters of MEDE.
(3) In the bearing fault identification stage of the proposed method, although a KNN model with high efficiency and few parameters was adopted, it had a lot of dependence on the labels of the data sample. That is, this classification process was equivalent to a supervised learning process. Hence, to get rid of the dependence of data labels and achieve the goal of unsupervised learning, in future work, we will adopt clustering algorithms (e.g., k-means, fuzzy c-means, or self-organizing-map clustering) to replace the KNN model to obtain bearing fault identification results.

## 6. Conclusions

This paper proposes a new bearing fault diagnosis method based on parameter adaptive variational mode extraction and multiscale envelope dispersion entropy. Simulation and experimental signal analysis are conducted to validate the effectiveness of the proposed method. Experimental results show that the proposed method has a higher identification accuracy than other combined methods mentioned in this paper. The prominent contributions and novelties of this paper are summarized as follows:

(1) An improved signal processing method named parameter adaptive variational mode extraction based on whale optimization algorithm is presented, which can overcome the problem of artificial selection of the key parameters (i.e., penalty factor and mode center-frequency) existing in the original variational mode extraction.
(2) An effective complexity evaluation method called multiscale envelope dispersion entropy is proposed for bearing fault feature extraction by integrating the advantages of envelope demodulation analysis and multiscale dispersion entropy.
(3) A bearing intelligent diagnosis method is developed by combining parameter adaptive variational mode extraction and multiscale envelope dispersion entropy.
(4) The experimental results and comparison analysis prove the effectiveness and superiority of the proposed method in identifying different bearing health conditions.

It should be pointed out that this paper focuses on the identification of single bearing faults, but the identification of compound bearing faults is not considered in the paper. Therefore, compound fault identification of rolling bearing will be regarded as the key emphasis in our future work, where advanced deep learning models (e.g., deep convolutional network [41,42] and variational autoencoder [43]) will be combined with PAVME to identify bearing fault patterns.

## Figures and Tables

**Table 1:** The evaluation indexes obtained by different methods.

Different Methods	Kurtosis	Correlation Coefficient	RMSE	Running Time (s)
PAVME	5.7742	0.7966	0.2684	6.6552
VME	4.9841	0.7314	0.3130	0.1682
VMD	5.1330	0.7630	0.2916	99.528
EMD	2.4602	0.4023	0.8139	0.3704

**Table 2:** Size parameters of bearings.

Bearing Type	Roller Diameter (mm)	Pitch Diameter (mm)	Number of the Roller	Contact Angle
LYC6205E	7.94	39	9	0°

**Table 3:** Bearing fault characteristic frequencies (Hz).

Rotating Frequency	Inner Race Fault	Outer Race Fault	Ball Fault	Cage Fault
*f_r_* = 24.5	*f_i_* = 132.7	*f_o_* = 87.8	*f_b_* = 57.7	*f_c_* = 9.76

**Table 4:** The detailed description of bearing datasets.

Condition Label	Bearing Fault Types	Number of Training Samples	Number of Testing Samples	Class Label
Condition 1	Normal	50	50	1
Condition 2	Inner race fault (IRF)	50	50	2
Condition 3	Outer race fault (ORF)	50	50	3
Condition 4	Ball fault (BF)	50	50	4

**Table 5:** The optimal combination parameters of PAVME for different bearing fault signals.

The Key Parameters	IRF	ORF	BF
The penalty factor *a*	1236	1520	1138
The mode center-frequency *f_d_*	4352	4943	5113

**Table 6:** Diagnosis results of combining PAVME and different entropies in case 1.

Different Methods	Identification Accuracy Obtained Using Different Methods (%)			
	Maximum	Minimum	Mean	Standard Deviation
PAVME and MEDE	100	99.50	99.90	0.2108
PAVME and MDE	95.00	94.00	94.50	0.3333
PAVME and MPE	88.50	87.50	88.05	0.3689
PAVME and MSE	92.50	91.50	92.15	0.3375

**Table 7:** Diagnosis results of combining different signal processing methods and MEDE in case 1.

Different Methods	Identification Accuracy Obtained Using Different Methods (%)			
	Maximum	Minimum	Mean	Standard Deviation
PAVME and MEDE	100	99.50	99.90	0.2108
VME and MEDE	97.00	96.50	96.85	0.2415
VMD and MEDE	98.00	97.50	97.85	0.2415
EMD and MEDE	95.50	95.00	95.25	0.2635

**Table 8:** Size parameters of testing bearing in case 2.

Bearing Type	Roller Diameter (mm)	Pitch Diameter (mm)	Number of the Roller	Contact Angle (°)
SKF6205-2RS	7.94	39.04	9	0

**Table 9:** The detailed description of bearing dataset in case 2.

Bearing Health Conditions	Rotating Speed (r/min)	Fault Size (inches)	Number of Training Samples	Number of Testing Samples	Class Label
Normal	1797	0	25	25	1
Inner race fault 1 (IRF1)	1772	0.007	25	25	2
Inner race fault 2 (IRF2)	1772	0.014	25	25	3
Inner race fault 3 (IRF3)	1772	0.021	25	25	4
Outer race fault 1 (ORF1)	1750	0.007	25	25	5
Outer race fault 2 (ORF2)	1750	0.014	25	25	6
Outer race fault 3 (ORF3)	1750	0.021	25	25	7
Ball fault 1 (BF1)	1730	0.007	25	25	8
Ball fault 2 (BF2)	1730	0.014	25	25	9
Ball fault 3 (BF3)	1730	0.021	25	25	10

**Table 10:** The optimal parameters of PAVME for different bearing fault signals in case 2.

Bearing Health Conditions	The Penalty Factor *a*	The Mode Center-Frequency *f_d_*
Inner race fault 1 (IRF1)	835	1973
Inner race fault 2 (IRF2)	799	1991
Inner race fault 3 (IRF3)	958	2397
Outer race fault 1 (ORF1)	944	1889
Outer race fault 2 (ORF2)	1023	2763
Outer race fault 3 (ORF3)	1049	2854
Ball fault 1 (BF1)	1024	1998
Ball fault 2 (BF2)	1061	2717
Ball fault 3 (BF3)	1018	1913

**Table 11:** Diagnosis results of combining PAVME and different entropies in case 2.

Different Methods	Identification Accuracy Obtained Using Different Methods (%)			
	Maximum	Minimum	Mean	Standard Deviation
PAVME and MEDE	100	99.60	99.96	0.1265
PAVME and MDE	94.80	94.40	94.48	0.1687
PAVME and MPE	86.80	86.00	86.52	0.2700
PAVME and MSE	91.60	90.80	91.32	0.3293

**Table 12:** Diagnosis results of combining different signal processing methods and MEDE in case 2.

Different Methods	Identification Accuracy Obtained Using Different Methods (%)			
	Maximum	Minimum	Mean	Standard Deviation
PAVME and MEDE	100	99.60	99.96	0.1265
VME and MEDE	97.20	96.80	96.88	0.1687
VMD and MEDE	95.60	95.20	95.48	0.1932
EMD and MEDE	94.40	94.00	94.24	0.2066

## Data Availability

The data used in this study are all owned by the research group and will not be transmitted.

## References

[1] Vamsi I., Sabareesh G., Penumakala P. (2019). Comparison of condition monitoring techniques in assessing fault severity for a wind turbine gearbox under non-stationary loading. Mech. Syst. Signal Process..

[2] Yan X., Liu Y., Xu Y., Jia M. (2021). Multichannel fault diagnosis of wind turbine driving system using multivariate singular spectrum decomposition and improved Kolmogorov complexity. Renew. Energ..

[3] Lei Y., Lin J., He Z., Zuo M. (2013). A review on empirical mode decomposition in fault diagnosis of rotating machinery. Mech. Syst. Signal Process..

[4] Chen J., Pan J., Li Z., Zi Y., Chen X. (2016). Generator bearing fault diagnosis for wind turbine via empirical wavelet transform using measured vibration signals. Renew. Energ..

[5] Li Y., Xu M., Wang R., Huang W. (2016). A fault diagnosis scheme for rolling bearing based on local mean decomposition and improved multiscale fuzzy entropy. J. Sound Vib..

[6] An X., Zeng H., Li C. (2016). Demodulation analysis based on adaptive local iterative filtering for bearing fault diagnosis. Measurement.

[7] Yan X., Liu Y., Jia M. (2020). A fault diagnosis approach for rolling bearing integrated SGMD, IMSDE and multiclass relevance vector machine. Sensors.

[8] Dragomiretskiy K., Zosso D. (2014). Variational mode decomposition. IEEE Trans. Signal Process..

[9] Liu S., Yu K. (2022). Successive multivariate variational mode decomposition based on instantaneous linear mixing model. Signal Process..

[10] Chen G., Yan C., Meng J., Wang H., Wu L. (2021). Improved VMD-FRFT based on initial center frequency for early fault diagnosis of rolling element bearing. Meas. Sci. Technol..

[11] Kostopoulos S. (2013). Bearing fault detection based on hybrid ensemble detector and empirical mode decomposition. Mech. Syst. Signal Process..

[12] Yu Y., Subhani M., Hoshyar A., Li J., Li H. (2020). Automated health condition diagnosis of in situ wood utility poles using an intelligent non-destructive evaluation (NDE) framework. Int. J. Struct. Stab. Dyn..

[13] Zhao H., Zuo S., Ming H., Wei L., Ling Y. (2018). A novel adaptive signal processing method based on enhanced empirical wavelet transform technology. Sensors.

[14] Liu Z., Jin Y., Zuo M., Feng Z. (2017). Time-frequency representation based on robust local mean decomposition for multicomponent AM-FM signal analysis. Mech. Syst. Signal Process..

[15] Zhang Y., Lv Y., Ge M. (2021). A rolling bearing fault classification scheme based on k-optimized adaptive local iterative filtering and improved multiscale permutation entropy. Entropy.

[16] Zheng Z., Xin G. (2019). Fault feature extraction of hydraulic pumps based on symplectic geometry mode decomposition and power spectral entropy. Entropy.

[17] Jiang X., Li S., Cheng C. (2016). A novel method for adaptive multiresonance bands detection based on VMD and using MTEO to enhance rolling element bearing fault diagnosis. Shock Vib..

[18] Yi C., Lv Y., Dang Z. (2016). A fault diagnosis scheme for rolling bearing based on particle swarm optimization in variational mode decomposition. Shock Vib..

[19] Nazari M., Sakhaei S. (2018). Variational mode extraction: A new efficient method to derive respiratory signals from ECG. IEEE J. Biomed. Health Inf..

[20] Pang B., Nazari M., Tang G. (2022). Recursive variational mode extraction and its application in rolling bearing fault diagnosis. Mech. Syst. Signal Process..

[21] Wang Z., Jia L., Kou L., Qin Y. (2018). Spectral kurtosis entropy and weighted SaE-ELM for bogie fault diagnosis under variable conditions. Sensors.

[22] Han M., Pan J. (2015). A fault diagnosis method combined with LMD, sample entropy and energy ratio for roller bearings. Measurement.

[23] Zanin M., Zunino L., Rosso O., Papo D. (2012). Permutation entropy and its main biomedical and econophysics applications: A review. Entropy.

[24] Wu D., Zhang S., Zhao H., Yang X. (2018). A novel fault diagnosis method based on integrating empirical wavelet transform and fuzzy entropy for motor bearing. IEEE Access.

[25] Kang B., Yong D. (2015). The maximum deng entropy. IEEE Access.

[26] Yu J., Cao J., Liao W., Chen Y., Lin J. (2017). Multivariate multiscale symbolic entropy analysis of human gait signals. Entropy.

[27] Azami H., Escudero J. (2018). Coarse-graining approaches in univariate multiscale sample and dispersion entropy. Entropy.

[28] Mahajan R., Morshed B. (2014). Unsupervised eye blink artifact denoising of EEG data with modified multiscale sample entropy, Kurtosis, and Wavelet-ICA. IEEE J. Biomed. Health Inf..

[29] Zheng J., Pan H., Yang S., Cheng J. (2018). Generalized composite multiscale permutation entropy and Laplacian score based rolling bearing fault diagnosis. Mech. Syst. Signal Process..

[30] Yan X., Xu Y., Jia M. (2021). Intelligent fault diagnosis of rolling-element bearings using a self-adaptive hierarchical multiscale fuzzy entropy. Entropy.

[31] Zhou F., Shen J., Yang X., Liu X., Liu W. (2020). Modified hierarchical multiscale dispersion entropy and its application to fault identification of rotating machinery. IEEE Access.

[32] Azami H., Rostaghi M., Abásolo D., Escudero J. (2017). Refined composite multiscale dispersion entropy and its application to biomedical signals. IEEE Trans. Biomed. Eng..

[33] Kannan V., Li. H., Dao D. (2019). Demodulation band optimization in envelope analysis for fault diagnosis of rolling element bearings using a real-coded genetic algorithm. IEEE Access.

[34] Mirjalili S., Andrew L. (2016). The whale optimization algorithm. Adv. Eng. Softw..

[35] Miao Y., Zhao M., Makis V., Lin J. (2019). Optimal swarm decomposition with whale optimization algorithm for weak feature extraction from multicomponent modulation signal. Mech. Syst. Signal Process..

[36] Dong Y., Liao M., Zhang X., Wang F. (2011). Faults diagnosis of rolling element bearings based on modified morphological method. Mech. Syst. Signal Process..

[37] Ye M., Yan X., Jia M. (2021). Rolling Bearing Fault Diagnosis Based on VMD-MPE and PSO-SVM. Entropy.

[38] Yan X., Jia M. (2019). Intelligent fault diagnosis of rotating machinery using improved multiscale dispersion entropy and mRMR feature selection. Knowl. Based Syst..

[39] Tian J., Morillo C., Azarian M., Pecht M. (2016). Motor bearing fault detection using spectral kurtosis-based feature extraction coupled with k-nearest neighbor distance analysis. IEEE Trans. Ind. Electron..

[40] Ferracuti F., Freddi A., Monteriù A., Romeo L. (2021). Fault diagnosis of rotating machinery based on wasserstein distance and feature selection. IEEE Trans. Autom. Sci. Eng..

[41] Xie C., Liu Y., Zeng W., Lu X. (2019). An improved method for single image super-resolution based on deep learning. Signal Image Video Process..

[42] Yu Y., Liu Y., Chen J., Jiang D., Zhuang Z., Wu X. (2021). Detection method for bolted connection looseness at small angles of timber structures based on deep learning. Sensors.

[43] Yan X., She D., Xu Y., Jia M. (2021). Deep regularized variational autoencoder for intelligent fault diagnosis of rotor-bearing system within entire life-cycle process. Knowl. Based Syst..

